# Editorial: Pediatric adrenal neoplasms

**DOI:** 10.3389/fendo.2023.1227835

**Published:** 2023-06-05

**Authors:** Christina Pamporaki, Antje Redlich, David Taïeb, Karel Pacak

**Affiliations:** ^1^ Department of Medicine III, University Hospital Carl Gustav Carus, Technische Universität Dresden, Dresden, Germany; ^2^ Pediatric Oncology Department, Otto-von-Guericke-University Children’s Hospital, Magdeburg, Germany; ^3^ Department of Nuclear Medicine, Aix-Marseille University, La Timone University Hospital, Marseille, France; ^4^ Section on Medical Neuroendocrinology, Eunice Kennedy Shriver National Institute of Child Health and Human Development, National Institutes of Health, Rockville, MD, United States

**Keywords:** pediatric adrenal tumors, pheochromocytoma, neuroblastoma, adrenal cortical carcinoma, metastatic disease

Adrenal neoplasms present unique diagnostic and management challenges in pediatric patients related to both their rare and often highly aggressive nature. Although significant advances have been made during the last decades in the diagnosis and treatment of pediatric tumors, there is still no improvement of mortality in pediatric patients with adrenal neoplasms. One possible explanation could be the underestimation of these tumors due to their rare nature and the consequent lack of pediatric-based diagnostic and treatment protocols. Indeed, variable genomic alterations, clinical and biochemical presentations of adrenal tumors among different patient age groups often render the application of adult protocols in pediatric patients inappropriate. For example, pediatric patients with pheochromocytomas/paragangliomas (PPGLs) show significantly higher prevalence of extra-adrenal, multifocal, recurrent, and metastatic tumors compared to adults. As described by Bechmann et al. these findings can be partially explained by the higher prevalence of cluster 1 pathogenic variants in children than adults that activate pseudohypoxia-signaling pathways, and in turn lead to less differentiated tumor phenotypes. Similarly, Ilanchezhian et al. illustrated that children with adrenocortical carcinomas (ACC) present with higher hereditary predisposition, and in particular with higher overexpression of TP53 and insulin-like growth factor (IGFR) 2 pathogenic variants compared to adults, reflecting again different developmental origins between the two groups. Finally, illustration of the genetic background seems to be key for the management of more common and benign adrenal neoplasms in children, as discussed extensively in the draft of Pitsava and Stratakis.

Despite advances in the elucidation of the developmental origins of adrenal neoplasms, rare pediatric adrenal tumors are often misdiagnosed in daily clinical practice. A typical example is the misdiagnosis of PPGLs and ACCs for the more common neuroblastomas, resulting in worse patient outcomes as described in the case series of Kuhlen et al. Indeed, Kuo et al. well outlined that a biopsy of a PPGL falsely mistaken for neuroblastoma can lead to catecholamine excess crisis, whereas of an ACC to a high risk of tumor spillage, both situations dangerous or detrimental to a patient. A detailed medical history and a proper clinical patient evaluation can assist in raising clinicians´ suspicion for rare adrenal tumors. ACCs in children are often functional, with most common manifestation that of virilization due to androgen excess followed by signs and symptoms of Cushing syndrome. Similarly, as indicated by Eisenhofer et al., signs and symptoms of catecholamine excess (e.g headache, palpitations, diaphoresis) should immediately raise suspicion of a PPGL in a patient with apparent working diagnosis of neuroblastoma, as neuroblastomas do not usually secrete catecholamines in sufficient amounts to justify such clinical manifestations. This is mainly due to the lower amounts of neurosecretory vesicles in the cytoplasm of neuroblastomas compared to PPGLs, as suggested by Mühlerthaler-Mottet et al., resulting in decreased catecholamine storage and rapid metabolism of catecholamines to their O-methylated metabolites, metanephrines.

After clinical evaluation, hormone profiles must be assessed in pediatric patients tested for adrenal tumors, in dedicated laboratories with appropriate analytical methods and reference intervals. Establishment of pediatric reference intervals may be though, challenging. Indeed, as illustrated by Eisenhofer et al., biochemists face difficulties in the establishment of pediatric reference intervals for plasma and urinary metanephrines, due to their dynamic changes during childhood and adolescence. Nevertheless, appropriate pediatric reference intervals are always essential to minimize false positive and negative results and laboratories should never use adult reference intervals for pediatric patients. Apart from analytical considerations, pre-analytical precautions should also be considered in the biochemical work up of patients with catecholamine producing tumors. Specifically, children should remain in supine position for at least 20 minutes before blood sampling for the measurements of plasma metanephrines, whereas blood should be ideally drawn *via* intravenous cannula. Such precautions are expected to minimize activation of the sympathetic nervous system, and thus false elevations of plasma normetanephrine concentrations.

Apart from diagnosis, disease stratification is also challenging among pediatric patients with adrenal neoplasms due to their heterogeneous nature, which can range from spontaneous remission of a relatively benign course to dangerous hormonal hyper-secretion or rapidly progressive metastatic disease. Thus, identification of reliable prognostic tools is essential. In this direction, apart from the role of overexpression of HIF-2A as predictor of more undifferentiated and aggressive phenotypes for catecholamine producing tumors elucidated by Bechmann et al., Lv et al. established that advanced tumor stage, lack of complete tumor resection and older age at diagnosis are independent predictors of poor prognosis for pediatric patients with adrenal malignancies. The above findings provide immediate guidance for stratification and further patient management in daily clinical practice. Finally, advances in molecular imaging have also contributed substantially to improved stratification of pediatric patients with adrenal neoplasms. As described by Fargette et al. (1) ^123^I^-^MIBG and ^68^Ga-DOTATATE PET/CT functional imaging are today routinely used for the staging of patients with neuroblastoma and PPGL respectively, whereas ^18^F-FDG PET/CT has been effectively applied for the preoperative stratification of pediatric patients with ACC.

Illustration of underlying cellular and molecular mechanisms has proven beneficial also in the field of therapeutics. The role of HIF-2A in the HIF/MYC signaling has led to introduction of HIF-2α inhibitors for the treatment of metastatic/locally aggressive Cluster-1-related PPGLs. Importantly, findings from the pre-clinical study of Pacak et al. (2) in mice, indicate that intratumoral immunotherapy may constitute an effective future approach for the enhancement of the immune response of pediatric patients with PPGL. Finally, accumulating evidence, including the latest report by Urquhart et al., show that temozolamide may be a preferable alternative to systemic chemotherapy with cyclophosphamide/vincristine/dacarbazine in children with metastatic PPGL. With regard to pediatric patients with ACC, although the initially promising targeted therapies with the IGF1R-inhibitors failed expectations, Ilanchezhian et al. indicated that kinase inhibitors such as pembrolizumab or cabozantinib show promising results for the treatment of patients with advanced adrenocortical tumors.

Despite the aforementioned advances, there is need for multidisciplinary approaches and uniformly defined international diagnostic and therapeutic strategies for improved patient management. A solid body of literature has advocated the implementation of multidisciplinary approach for adherence to clinical guidelines, and improvement of patient outcomes. Indeed, findings from the study of Uttinger et al. indicate that surgical interventions of pediatric patients with adrenal neoplasms in high-volume referral centers is expected to minimize postoperative morbidity and increase treatment success rates. Finally, multidisciplinary approaches facilitate entry into transition and long-term surveillance programs, both crucial for the improvement of patient quality of life and the reduction of long-term mortality. Nevertheless, and despite the progress achieved during the last decades, transition care seems to be fragmented into mainstream oncology. Hence, future efforts should be focused on strengthening service coordination, infrastructure, resources, and finally, proper education of pediatric and adult endocrine oncologists in transition medicine.

## Author contributions

CP has written the manuscript. All authors revised the article and approved the submitted version.
